# Acinic Cell Carcinoma of the Breast: A Case Report and Review of Literature

**DOI:** 10.7759/cureus.51427

**Published:** 2024-01-01

**Authors:** Ihab S Atta

**Affiliations:** 1 Pathology Department, Faculty of Medicine, Al-Baha University, Al-Baha, SAU; 2 Pathology Department, Faculty of Medicine, Al-Azhar University, Cairo, EGY

**Keywords:** dog-1, etv6-ntrk3, breast cancer, amylase, alcian blue, acinic cell carcinoma

## Abstract

Acinic cell carcinoma (ACC) is an exceedingly rare type of triple-negative breast cancer (TNBC). We are reporting a case of a 46-year-old female patient who presented with a palpable lump in her left breast not associated with pain, pruritis, or change of skin color. An open biopsy revealed a mass of about 20 x 25 mm of fleshy, white tan with a lobular configuration and necrosis. The histopathological examination revealed cells with cytoplasmic granularity arranged in a microglandular pattern and a solid pattern, and the case was initially reported as ACC. The most remarkable feature was the presence of small and large, brightly eosinophilic cytoplasmic granules, and some cells are clear or multivacuolated, resembling lipoblasts. Cellular pleomorphism and anaplasia are very mild, and the mitotic activity was very low. The tumor showed a scant and vascularized stroma in the area of hyalinization. Small clusters of lymphoid infiltration in the stroma were seen. Histochemical stains revealed that the acinar cells in ACC contain abundant diastase-resistant, periodic acid Schiff (PAS)-positive cytoplasmic granules. Mucicarmine and Alcian blue were negative. The immunohistochemistry workup revealed that the case was positive for discovered on gastrointestinal stromal tumors-1 (DOG-1) and the positivity pattern ranged from apical membranous, cytoplasmic, and complete membranous. In addition, the tumor cells were positive for low-molecular-weight cytokeratin, carcinoembryonic antigen (CEA), and epithelial membrane antigen (EMA). The FISH workup for the ETV6-NTRK3 fusion was negative, arguing against secretory carcinoma (SC). A diagnosis of acinar cell carcinoma of the breast is very rare, and the presence of cytoplasmic granules is helpful for its diagnosis. In the absence of these granules, the diagnosis is very difficult, and other diagnoses will be put in the differential diagnosis, particularly SC, lactating adenosis, and microglandular adenosis. Immunohistochemical and histochemical stains and genetic workups will support the diagnosis of ACC.

## Introduction

The incidence of acinic cell carcinoma (ACC) in the breast is low, and the actual incidence is still unidentified. The WHO classified the ACC as one of the breast cancers of other invasive carcinoma subtypes [1]. It is considered one of the triple-negative breast cancers (TNBCs) [2]. The incidence of ACC in the breast is very rare, and its resemblance to secretory carcinoma (SC) makes its diagnosis very difficult. In addition, WHO categorized this tumor as one of the "other invasive carcinoma subtypes" owing to its rarity, morphologic, and immunohistochemical characteristics.

By reviewing and analyzing what was recorded regarding the diagnosis of ACC from 2022 down until the first case was recorded in 1996, the following was found: about 68 cases were diagnosed as primary ACC of the breast between 1996 and 2022, and the age group ranged from 23 years up to 80 years [3-5]. Among these cases, a male case was diagnosed by Shimao et al. (1998) [5] aged 23 years in the left breast, and ultrasonography showed a clear marginal cystic mass composed of hypoechoic intracystic fluid and a hyperechoic intracystic tumor. The maximum number of reported cases was seen in 2014 and 2015 (13 and 9 cases, respectively) [6-9]. Most of the reported cases were microglandular, solid, or a mixture of both [10-12], three cases were papillary [13,14], one case was microfollicular [13], and one case showed a clear cell histomorphology [8].

Mammography revealed varieties of interpretations ranging from no abnormalities detected [11], occult carcinoma [15], a small oval radiopaque mass [16], a thickening with microcalcifications [17], a firm nodular lesion, a well-demarcated mass without microcalcifications to lobulated mass with poorly defined margins, without microcalcification, to a mass with well-defined margins with scattered granular calcifications [8]. Ultrasonography ranged from no abnormalities detected to a hypoechoic nodule with a smooth surface. MRI revealed heterogeneous segmental non-mass-like enhancement [3,8].

The aim of this case introduction is to identify the new case of ACC in the female breast, describe its histopathological, histochemical, immunohistochemical, and genetic workup, and correlate the findings of the present case with other previously reported cases.

## Case presentation

A 46-year-old female patient presented with a palpable lump in her left breast not associated with pain, pruritis, or change of skin color. The patient noticed this lump three months prior and noticed a slight increase in size over the last month. A physical examination confirmed the presence of a lump, and a mammography was required. Mammography showed a nodular, firm lesion with the presence of scattered microcalcifications. Ultrasonography revealed a hypoechoic nodule with a smooth surface. MRI revealed heterogeneous segmental, non-mass-like enhancement.

An open biopsy revealed a mass of about 20 x 25 mm of fleshy, white tan with a lobular configuration and necrosis. The histopathological examination revealed cells with cytoplasmic granularity arranged in a mixture of solid and trabecular pattern patterns, and the case was initially reported as ACC.

The most remarkable feature was the presence of small and large, brightly eosinophilic cytoplasmic granules. Small numbers of cells showed vacuolated or clear cytoplasm. Secretory material revealing a lower degree of eosinophilia was seen in most tubular structures. No vascular invasions were seen.

**Figure 1:**
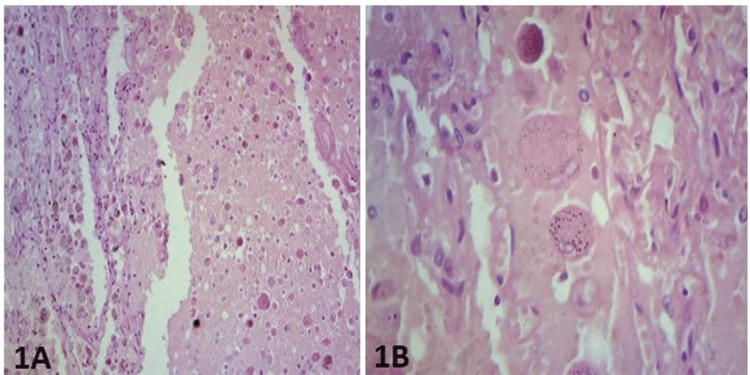
(1A) The mastectomy specimen shows acinar cells with cytoplasmic granules (hematoxylin & eosin stain, x200). (1B) The granules are more prominent and dot-like (hematoxylin & eosin stain, x400)

In the mastectomy specimen, there was an irregular fleshy area measuring 2.5 x 1.6 x 2 cm in all dimensions. Histopathologic examination revealed that a mixture of solid and trabecular patterns was the most common. Many of the cells contained granular cytoplasm, and some cells were clear or multivacuolated, resembling lipoblasts (Figure 1A-1B).

**Figure 2:**
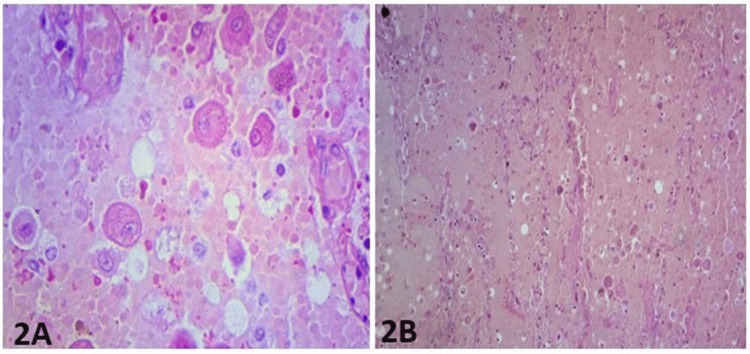
(2A) A figure shows the cells with eccentric nuclei and prominent nucleoli, eosinophilic cytoplasmic granules admixed with some cells that show clear or multivacuolated resembling lipoblasts (hematoxylin & eosin stain, X400). (2B) A section showed that these cells are set in the background of hyalinized stroma (hematoxylin & eosin stain, x200)

In some areas, the cells had deeply eosinophilic cytoplasm. Cellular pleomorphism and anaplasia were very mild, with low mitotic activity. The tumor showed hyalinized stroma in some areas (Figure 2A-2B).

**Figure 3:**
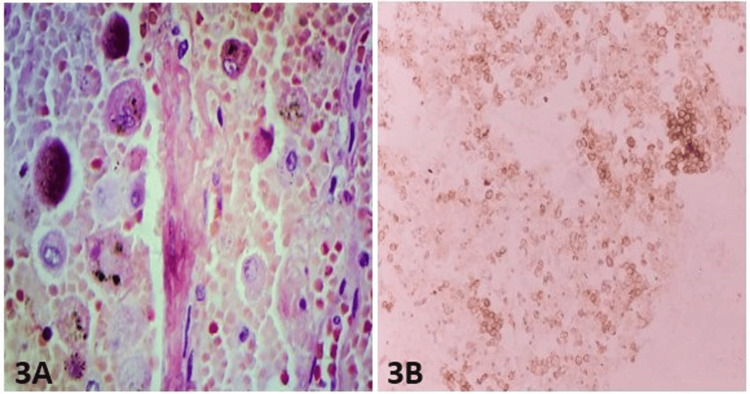
(3A) A figure shows acinar cells containing abundant diastase-resistant, PAS-positive cytoplasmic granules (PAS-D stain) (PAS-D, x400). (3B) This section reveals that the case is immunoreactive for DOG-1 (DOG-1 stain, X200)

Small clusters of lymphoid infiltration in the stroma were seen. Histochemical stains revealed that the acinar cells in ACC contain abundant diastase-resistant, periodic acid Schiff (PAS)-positive cytoplasmic granules. Mucicarmine and Alcian blue are negative. The immunohistochemistry workup revealed that the case was positive for discovered on gastrointestinal stromal tumors-1 (DOG-1) and the positivity pattern ranged from apical membranous, cytoplasmic, and complete membranous (Figure 3A-3B).

In addition, the acinar cells were positive for low-molecular-weight cytokeratins (CK5/6), carcinoembryonic antigen (CEA), and epithelial membrane antigen (EMA). S100 protein and amylase showed focal cytoplasmic positivity. In addition, the proliferating activity marker Ki-67 labeling index was low (<5%). FISH revealed no presence of ETV6/NTRK3 fusion.

The patient underwent a mastectomy and axillary lymph node dissection, revealing 15 negative nodes. Postoperatively, the patient was treated with chemotherapy followed by radiotherapy, and at a one-year follow-up, the patient was disease-free and discharged from further medical care from this standpoint.

## Discussion

Reviewing the histological background of ACC

ACC demonstrates a group of growth patterns such as solid, microglandular, or pseudolobular [16], microlobular [18], cystic, cribriform [19], trabecular [20], microcystic [10], follicular, and papillary cystic [13,14]. The solid and microcystic patterns are the most common and are often seen together [7,16,18]. In a well-differentiated ACC, the acinar cells predominate; however, in a few tumors, they are seen in the minority [21]. In addition, cellular pleomorphism and anaplasia are usually absent with low mitotic activity [22,23].

The tumor has a scant and vascularized stroma; however, it may be dense and hyalinized. Furthermore, a prominent lymphoid infiltration in the stroma is seen in most cases which may lead to misdiagnosis of lymph node metastasis. In addition, many ACCs show a prominent lymphoid component with a germinal center reminisce to that of Warthin’s tumor [24]. ACC may display follicular or glandular patterns [11].

The microcystic pattern is commonly seen in which small cystic spaces are present, which may be empty or filled with basophilic or eosinophilic material. In papillary-cystic patterns, there are cystic spaces of variable sizes with papillary projections arising from the cyst wall into lumina, which are often filled with proteinaceous eosinophilic material. Sometimes some epithelial proliferation is seen freely floating in the cyst space [12].

Some cases of ACC show secondary changes that may happen either following fine needle aspiration or spontaneously, such as cystic degeneration, infarction, hemorrhage, lipogranulomatous reaction, cholesterol clefts, chronic inflammation, and fibrosis [10].

The acinar cells in ACC contain abundant diastase-resistant, PAS-positive cytoplasmic granules. Mucicarmine and Alcian blue are usually negative [2,18]. The immunohistochemistry workup revealed that ACC is DOG-1 positive, and the positivity pattern ranged from apical membranous, cytoplasmic, to complete membranous [3,18,25]. Other positive markers for acinar cells include low-molecular-weight cytokeratins, CEA, and EMA [2,26]. Focal amylase immunoreactivity may be seen in a small percentage of cases [9]. In addition, proliferating activity markers such as the Ki-67 labeling index are usually low [2,26] As reported, the primary ACC is a rare entity of breast cancer and, in most cases, is categorized as triple-negative cancer. There is strong evidence that ACC does not always have a good prognosis, and additional research is necessary to recognize the forecasters of poor outcomes. Weaver et al. [18] reported that ACC is a triple-negative steroid receptor with HER-2/neu status and positive for DOG-1 and Gata-binding protein 3 (GATA3). However, rare cases of ACC of less than 10% show positivity for ER and PR [12,25]. Androgen receptor (AR) is also found to be positive in 10% [27]. Although these studies were performed prior to routine application of DOG-1 stain and some of the cases may instead represent apocrine carcinomas, a variant of ductal carcinoma NOS also shows prominent cytoplasmic granules.

Transformation to high grade is nearly predicted in 10% of well-differentiated ACCs, frequently in older patients with recurrent or long-standing tumors. It is often characterized by an interval of rapid growth, pain, fixation to surrounding tissues, an increased growth rate, and a poor outcome [28]. Occasionally, high-grade morphology may be observed at the initial presentation and consists of areas of conventional (low-grade) carcinoma intermingled with areas of high-grade carcinoma. The high-grade carcinoma is composed entirely of poorly differentiated or even undifferentiated cells with trabecular or solid growth patterns associated with high abnormal mitoses and necrosis [28]. The differential diagnosis of ACC is SC, lactating lobule, and microglandular adenosis [29-36].

SC is defined as a low-grade salivary gland neoplasm. It consists of multiple lobules bounded by fibrotic stroma. The lobules demonstrate varied growth patterns, including microcystic, solid, macrocytic, tubular, papillary, and cribriform. Occasionally, multiple patterns may be present within the tumor, even within the lobules. The tumor cells show bland morphology in which uniform round to oval vesicular nuclei and inconspicuous nucleoli are seen. The cytoplasm may be eosinophilic/granular or vacuolated. The high-grade cytologic features and increased mitoses are not seen [29,30].

SC is usually positive for cytokeratin 7, low-molecular-weight cytokeratin, signal transducer and activator of transcription 5A (STAT5a), vimentin, and S-100 protein. Gross cystic disease fluid protein 15 (GCDFP-15) and mammaglobin are also positive. DOG-1 is negative. Myoepithelial and basal cell markers are usually not expressed [30].

The differential diagnosis of SC includes zymogen-poor ACC, low-grade adenocarcinoma, low-grade salivary duct carcinoma, and cystadenocarcinoma NOS. The most interesting entity to differentiate the zymogen-poor ACC from SC. The positivity of DOG-1, along with the absence of common chromosomal rearrangement ETV6-NTRK3 fusion, assists in the identification of zymogen-poor ACC [31]. The SC is considered a low-grade carcinoma with a favorable prognosis. The risk of local recurrences is 15%, lymph node metastases are 20%, and distant metastases are 5%. The mortality rate is observed in 6% to 13% of cases diagnosed as SC [32].

Features confirming SC involve a lack of basophilic cytoplasmic zymogen granules, along with strong immunoreactivity for S-100 and mammaglobin, and the presence of ETV6-NTRK-3 gene fusion resulting from genetic t (12;15) (p13; q25) translocation. This genetic rearrangement is not confined to SCs of the salivary glands and breasts but has also been seen in other tumors such as cellular mesoblastic nephroma, congenital fibrosarcoma, and, in some cases, acute myeloid leukemia [31]. In a subcategory of SC cases, the ETV6 is rearranged on FISH; however, the ETV6-NTRK3 fusion transcript is not demonstrable by RT-PCR, signifying heterogenous breakpoints or another partner gene [32].

The lactating lobule revealed positivity for lysozyme and showed benign features, prominent cytoplasmic vacuoles, and abundant secretory material. The epithelial cells may have a hobnail or bulbous appearance, and the myoepithelial cells are inconspicuous [33].

Microglandular adenosis is a disordered proliferation of small open glands containing bland epithelial cells with intraluminal eosinophilic secretions. The basement membrane is intact in the absence of myoepithelium. These glands infiltrate the fibrous and adipose stroma without a stromal response. Immunohistochemistry revealed positivity for S-100 and negativity for estrogen and progesterone receptors (ER, PR) [34-36].

The differential diagnosis of ACC of the breast with high-grade transformation comprises adenoid cystic carcinoma with high-grade transformation, which revealed a typical tubular-cribriform growth pattern, adding to the presence of inner p63-negative ductal cells and outer p63-positive basal/myoepithelial cells [35].

Analysis of the previously reported cases of ACC

Analysis of the previously reported cases from 2022 down to 1996 revealed the following:

In 2022, three cases were reported by Sarsiat et al. [21] and Yu et al. [22], and Sarsiat et al. [21] reported a case of a 59-year-old presented with a mass in the right breast, in which mammography revealed an ill-defined mass and showed a solid and microglandular growth pattern on microscopic examination. Yu et al. [22] reported two cases aged 42 and 49 years. The first was detected during screening, and the second was occult cancer as seen by a mammography. Both showed solid and microglandular in addition to pseudolobular in the second case.

In 2021, Weaver et al. [18] reported a case of a 40-year-old with solid, microglandular, and pseudolobular, and ultrasonography revealed a well-defined, lobulated, solid lesion with characteristics of a fibroadenoma. In 2018, Sen et al. [11] reported a case of a 41-year-old with acinar and solid patterns, and no abnormalities were detected by ultrasonography and mammography.

In 2017, four cases were reported by Li et al. [19] and Kim et al. [36]. Li et al. [19] reported a case of a 52-year-old with a left breast mass showing a cystic and cribriform pattern, while Kim et al. [36] reported three cases on the left side without radiological details.

In 2016, five cases of ACC were reported. Kawai et al. [20] stated the case of a 49-year-old, and mammography showed a lobulated mass with poorly defined margins without microcalcification. Solid-trabecular and acinar findings were reported. Xu et al. [16] reported a case of a 41-year-old with a mass on the right breast, and mammography revealed a small oval radiopaque mass, and acinar-glandular and solid patterns were reported. In addition, Sherwell-Cabello et al. [37] reported a case with a microglandular pattern without radiological details. Conlon et al. [15] reported two cases aged 47 and 49. Both are occult in mammography, as are microglandular and solid patterns histologically.

In 2015, nine cases were reported. Guerini-Rocco et al. [7] reported eight cases ranging in age from 34 to 70 years, and one of these cases revealed a clear cell pattern, while others were microglandular. Piscuoglio et al. [6] reported a case of a 45-year-old with a microglandular pattern.

In 2014, 13 cases were diagnosed with ACC. Of these 100 cases reported by Zhong et al. [8], with an age range of 43-61 years, one case of a 26-year-old with an acinic-glandular and solid pattern was reported by Limite et al. [9], and one case of a 38-year-old with a microglandular pattern was reported by Zhao et al. [38].

In 2013, seven cases of ACC were reported. Osako et al. [14] reported three cases of 37, 46, and 50 years. The first was microglandular, the second was microglandular and solid, and the third was papillary, microglandular, solid, and cystic. In addition, Falleti et al. [17] reported a case of 58 presented with a periareolar mass, and mammography revealed a thickening with microcalcifications, and the histological pattern was a mixture of solid, microglandular, and microacinar. Other reported cases were documented by Shingu et al. [25], Winkler et al. [39], Ripamonti et al. [40], and Sakuma et al. [10]. Shingu et al. [25] reported a case of a 41-year-old, and mammography revealed the focal asymmetric density of the breast, which was supported by the presence of a heterogeneous hypoechoic mass with ill-defined margins in ultrasonography and by the presence of a high-intensity mass in MRI. No predominant patterns were seen and a mixture of solid, trabecular, and microglandular was present.

Winkler et al. [39] reported a case of a 56-year-old, and MRI revealed heterogeneous segmental non-mass-like enhancement extending toward the nipple in a triangular configuration. A solid pattern was seen histologically. Ripamonti et al. [40] reported a case of a 44-year-old, and an MRI showed a solid mass with pushing borders and microglandular histology. Sakuma et al. [10] reported a case of a 61-year-old, and mammography revealed dense, inhomogeneous parenchyma; ultrasonography showed a hypoechoic, irregularly shaped mass with ill-defined borders; and solid and microcystic were seen histologically.

In 2012, Choh et al. [41] reported a case of a 79-year-old with a solid pattern. In 2011, Chang et al. [27] reported a case of a 39-year-old with no abnormalities detected either in ultrasonography or mammography, and acinar/glandular and solid patterns were seen. In addition, Huo et al. [42] reported three cases aged 30, 40, and 51, which revealed microglandular, solid, and focally microcystic. In 2010, Stolnicu et al. [12] reported a case of a 79-year-old, and radiology revealed malignant features and varied patterns such as solid, microcystic, microglandular, and trabecular. In 2009, one case of a 62-year-old was reported by Matoso et al. [43], which revealed the presence of zymogen-like secretory granules and amphophilic cytoplasm.

In 2007, one case of an 80-year-old was reported by Tanahashi et al. [4], which showed a well-demarcated mass without microcalcifications on mammography, and an acinar pattern was seen. In 2004, another case was reported by Peintinger et al. [44] aged 36 years old with solid and microglandular patterns. In 2003, Kahn et al. [45] reported a case of a 56-year-old with a solid pattern.

In 2002, three cases of 20-, 59-, and 61-year-olds were reported by Hirokawa et al. [13], and mammography and ultrasonography revealed a solid lesion and an irregular-shaped mass with microcalcifications. Histologically, papillary/cystic, microcystic, and follicular microfollicular patterns were observed. In addition, Coyne et al. [46] reported a case of a 49-year-old with microglandular patterns.

In 2000, Damiani et al. [26] reported six cases of 35-, 42-, 55-, 63-, 64-, and 80-year-olds with solid and microglandular patterns. Furthermore, Schmitt et al. [47] reported a case of a 79-year-old, and mammography revealed a round and well-circumscribed lesion, and the solid pattern was predominant.

In 1998, one case of a 23-year-old male was reported in a male patient by Shimao et al. [5]. A radiological workup revealed a clear marginal cystic mass composed of hypoechoic intracystic fluid; a hyperechoic intracystic tumor was seen through ultrasonography; and a solid pattern was seen.

In 1996, Roncaroli et al. [48] reported the first case of a 42-year-old, and mammography revealed a mass with well-defined margins with scattered granular calcifications was seen in mammography, and a solid pattern was the predominant pattern. The previously reported cases are summarized in Table 1.

**Table 1:** This table summarizes the previously reported cases of ACC of both male and female breasts starting from 2022 down to the time of the first case which was diagnosed in 1996

Author	Pattern	Mammography/radiological workup	Gender	Age and number of cases
Roncaroli et al. (1996) [48]	Solid	Well-defined margins with scattered granular calcifications	Female	42
Shimao et al. (1998) [5]	Solid	A clear marginal cystic mass composed of hypoechoic intracystic fluid and a hyperechoic intracystic tumor	Male	23
Schmitt et al. (2000) [47]	Solid	A round and well-circumscribed lesion	Female	79
Damiani et 61 al. (2000) [26]	Solid and microglandular	A round and well-circumscribed lesion	Females	35-80 (6 cases)
Coyne et al. (2002) [46]	Microglandular	Not done	Female	49
Hirokawa et al. (2002) [13]	Papillary cystic, follicular	Solid lesion, An irregular-shaped mass with microcalcifications.	Females	20-61 (3 cases)
Kahn et al. (2003) [45]	Solid	Not available	Female	56
Peintinger et al. (2004) [44]	Solid and microglandular	Not available	Female	36
Tanahashi et al. (2007)[4]	Acinar	A hypoechoic nodule with a smooth surface, mammography showed a well-demarcated mass without microcalcifications.	Female	80
Matoso et al. (2009) [43]	Solid pattern	Not available	Female	62
Stolnicu et al. (2010) [12]	Solid, microcystic, microglandular, and trabecular	Signs of malignant features	Female	79
Huo et al. (2011) [42]	microglandular, solid, and focally microcystic	Not available	Females	30-51 (3 cases)
Chang et al. (2011) [27]	Acinar/glandular and solid	No abnormalities	Female	39
Choh et al. (2012) [41]	Solid	Not available	Female	79
Sakuma et al. (2013) [10]	Solid and microcystic	Dense, inhomogeneous parenchyma; Ultrasonography: A hypoechoic, irregularly shaped mass with ill-defined borders	Female	61
Ripamonti et al. (2013) [40]	Microglandular	A solid mass with pushing borders	Female	44
Winkler et al. (2013) [39]	Solid	Heterogeneous segmental non-mass-like enhancement extending toward the nipple in a triangular configuration	Female	56
Shingu et al. (2013) [25]	Solid, trabecular, and microglandular	Focal asymmetric density of the breast, ultrasonography showed a heterogeneous hypoechoic mass with ill-defined margins, MRI revealed high-intensity mass, but no intraductal dissemination	Female	41
Osako et al. (2013) [14]	Papillary, solid, microglandular	Not available	Females	37-50 (3 cases)
Falleti et al. (2013) [17]	Solid, microglandular and microacinar	A thickening with microcalcifications, firm, nodular lesion	Female	58
Zhao et al. (2014) [38]	Microglandular	Not available	Female	38
Limite et al. (2014) [9]	Acinar, solid	Not available	Female	26
Zhong et al. 26 (2014) [8]	All patterns	Not available	Females	43-61 (11 cases)
Piscuoglio et al. (2015) [6]	Microglandular	Not available	Female	45
Guerini-Rocco et al. (2015) [7]	Clear cell, microglandular.	Not available	females	34-70 (8 cases)
Conlon et al. 14 (2016)	Microglandular, solid	Occult	Females	47-49 (2 cases)
Sherwell-Cabello et al. (2016) [37]	Microglandular	Not available	Female	40
Xu et al. (2016) [16]	Acinar-glandular and solid	Small oval radiopaque mass and acinar-glandular and solid	Female	41
Kawai et al. (2016) [20]	Solid-trabecular and acinar were reported.	Lobulated mass with poorly defined margins, without microcalcification	Female	49
Kim et al (2017) [36]	Microglandular, solid	Not available	Females	40-50 (3 cases)
Li et al. (2017) [19]	Cystic and cribriform	Not available	Female	52
Sen et al. (2018) [11]	Acinar and solid	Not available	Female	41
Weaver et al. (2021) [18]	Solid, microglandular, pseudolobular	A well-defined, lobulated, solid lesion with characteristics of a fibroadenoma	Female	40
Yu et al. 3 (2022) [22]	Solid and microglandular, pseudolobular	Occult and the second during ordinary screening	Females	42-49 (2 cases)
Sarsiat et al. (2022) [21]	Solid and microglandular	Ill-defined mass	Female	59

As reported, the primary ACC is a rare entity of breast cancer and, in most cases, is categorized as triple-negative cancer. There is strong evidence that ACC does not always have a good prognosis, and additional research is necessary to recognize the forecasters of poor outcomes.

Clinically, it is considered a low-grade tumor even if unfavorable prognostic indicators such as steroid hormone receptor negativity and a high mitotic rate exist [44]. Primary ACC of the breast has good behavior in most reported cases, despite metastases in axillary lymph nodes that have been reported in five cases [26,42,46,48]. About 12% of all ACCs develop metastatic disease [49]. A poor prognosis of ACC has been reported, especially with a high grade of lymph node metastases [27]. Therefore, lymph nodes should be identified anatomically and examined properly, especially axillary sentinel lymph nodes. The sentinel nodes are the first few nodes to which ACC metastasizes [50].

Limitations of the study

In the present case, further data for the genetic workup is not available, and the genetic workup that revealed no ETV6-NTRK3 fusion was obtained only from the referral letter.

## Conclusions

The present case was diagnosed as ACC according to histopathological and immunohistochemical workups. ACC of the breast is very rare and put in the differential diagnosis with the SC. ACC contains abundant diastase-resistant and PAS-positive cytoplasmic granules, and the absence of these granules makes its diagnosis very difficult. In addition, ACC is usually negative for mucicarmine and alcian blue and positive for DOG-1. ACC is one of the TNBCs.

Clinically, it is considered a low-grade tumor, even though prognostic indicators are unfavorable. Primary ACC of the breast has good behavior in most reported cases. A poor prognosis of ACC has been reported, especially with a high grade of lymph node metastases. Thus, lymph nodes should be identified anatomically and examined properly, especially axillary sentinel lymph nodes. The sentinel nodes are the first few nodes to which ACC metastasizes. Clinicians should take into consideration the transformation of ACC into a high grade. Thus, periodic examinations, close monitoring, and a proper management plan should be applied. In addition, a genetic study should be done on those cases to confirm the diagnosis, along with histochemical and immunohistochemical workups.
